# Thank you to our reviewers

**DOI:** 10.1017/cts.2026.10688

**Published:** 2026-02-17

We are indebted to the expert referees who have volunteered their time to review submissions for the Journal of Clinical and Translational Science in 2025. The editors, authors, and readers are immensely grateful for their thoughtfulness and expertise that have served our journal well.

Maryam Abdallah

Mohamed Abou-el-Enein

Stacey Adam

Reuben Adatorwovor

Tiwaladeoluwa Adekunle

Mohammad Adibuzzaman

Andrew Admon

Ojo Akinlolu

Praveen Akuthota

Christina Alaniz

Philip Alberti

John Alexandar

Karen Alexander

Sallie Allgood

Michael Allon

Elizabeth Alonso

Natalie Altschuck

Laura Amendola

Andrew Anderson

Emily Anderson

Michele Peake Andrasik

James Andrews

Erdembileg Anuurad

Kat Aribindi

Jennifer Armstrong

David Asai

Kelly A. Aschbrenner

LauraEllen Ashcraft

Mona AuYoung

Charletta A. Ayers

Kara Ayers

Andrew Balas

Bijal Balasubramanian

Christian Balbin

Michael Eliot Bales

Lauren Balmert

Shweta Bansal

Claudia Baquet

Catherine Barnes

Nadine J. Barrett

Tara Bautista

Christopher Becker

Gordon R. Bernard

Carolyn Best

Eric Beyer

Kush Bhatt

Barbara Bierer

Leslie Biesecker

Carolyn Bishoff

Bradley Bishop

Nicquet Blake

Arthur Blank

Lauren Balmert Bonner

Kostiantyn Botnar

Kristopher Bough

Allan R. Brasier

Justin Scott Brathwaite

John Bridges

Guy Brock

Tabetha A. Brockman

Jordan Brown

Susan Cummings Budinger

Michael A. Bushey

Wade Bushman

Tatiana Bustos

Angela Byars-Winston

Renee B. Cadzow

Karen Calhoun

Robert M. Califf

Cliff Callaway

Carrie Cameron

Kenzie A. Cameron

Ramon Cancino

Bernadette Capili

Bobbi Carothers

Olveen Carrasquillo

Jennifer Carrera

Rickey Carter

Lori Carter-Edwards

Cameron Casey

Jon Casey

Shannon Casey

Yelba Castellon-Lopez

Patricia Chalela

Jack Chang

Ranee Chatterjee

Hongyu Chen

Alex C. Cheng

Neeraj Chhabra

Helen H.L. Chiu

Hwayoung Cho

Summer Choudhury

Kurt Christensen

Yong-Hee Patricia Chun

Terry Church

Barbara Ciastek

Janet T. Civian

Nichelle L. Cobb

Cemil Colak

Stephen Phillip Colegate

Barry Coller

Todd B. Combs

Lisa Connally

Nicola Conran

Dan Michael Cooper

Jillian Cotter

Linda Cottler

Jennifer Croker

Ted Cross

Sheila Crowell

Marina Cuchel

Colleen Cuddy

Mollie Cummins

Jennifer Cunningham-Erves

Geoffrey Curran

Rosalina Das

Gaurav Dave

Suja Davies

Melinda Marie Davis

Ganisher Davlyatov

Sonsoles De Lacalle

Peter Degen

Guilherme DelFiol

Kimberly Ellen Demirhan

Colin A. Depp

Deborah DiazGranados

Mirella Diaz-Santos

Kirsten Dickins

Lisa DiMartino

Sarah Donahue

Gerardine Doyle

Ann M. Dozier

Karen DSouza

Xin Du

James M. DuBois

Christopher Duffrin

James Dunham

Ysabel Duron

Amy Dworkin

Carrie Dykes

Brenda Eakin

Dixie Ecklund

Elizabeth Eckstrom

Milton Mickey Eder

Alyson G. Eggleston

Jordan Eidlisz

Howard Eisenson

Eric Eisenstein

Omar El Kawkgi

Angela R. Elam

Vicki L. Ellingrod

Helena Ellis

Philip Empey

Felicity T. Enders

Kyle T. Enriquez

Fayron Epps

Megan L. Falsetta

Jungwei W. Fan

Yilu Fang

Paolo Fantozzi

Elissa Faro

Moshtagh R. Farokhi

Maryam Farzad

Jessica M. Faupel-Badger

Josh Fehrmann

Rebecca Feinstein

Brandi Fink

Karen R. Fisher

Meredith B. Fitz-Gerald

Lisa A. Fleming

Konadu Fokuo

James Foran

Daniel Ford

Deborah M. Fournier

Andrew T. Fox

Stephanie Freel

Christopher Frei

Jessica M. Fritter

Al’ona Furmanchuk

Alvaro Gaitan

Emily Gallagher

Tanja Gangarova

Martha Garcia

Max Geraedts

Sue Giancola

Zachary Giano

Kevin Gibbs

Ralph A. Gigliotti

Murray Gillies

Liane Ginsberg

Cristina Godoy

Judith D. Goldberg

Stacey M. Gomes

Emma Goodwin

Bruce Gordon

Tyler Gorham

Urszula Górska-Klimowska

Steven C. Grambow

Gillian Gresham

Allison Grimes

Lourdes Guerrero

Jia-Wen Guo

Yi Guo

Melissa Haendel

Nathaniel Hafer

Alex Hall

Mark Hall

Candace Bright Hall-Wurst

Scott Halpern

Alexandra Hanlon

Md Rejuan Haque

Valerie Harder

Christopher A. Harle

Joanne Harmon

Alexandra E. Harper

Diane Harper

Daniel Harris

Lisa Harrison

Anuradha Hashemi

Amr Hassan

Larry W. Hawk, Jr.

Mary Ryan Hawkins

Sarah Haynes

Helen Hazuda

Xing He

Vahe Heboyan

Shirley L.T. Helm

Stuart Henderson

Adrian F. Hernandez

Chloe Hery

Keith Herzog

Rachel Hess

Patrice Hicks

Camille Hochheimer

Philip Hockberger

Samantha Holden

Young-Rock Hong

Matt Honore

Thomas J. Hoogeboom

Sara Horton

Carol Howe

Verónica Hoyo

David Huang

Yen-Ming Huang

Tim Huerta

Joe Hunt

Nan Huo

Phillip Ianni

David H. Ingbar

Ingrid Rivera Iñiguez

Tiffany Israel

Richard F. Ittenbach

Rebekah Jacob

Laura Jacobus

Carlos Jaén

Andrew J. James

Laura P. James

Xia Jing

Elizabeth Johnson

Brenda Joly

Carolynn Thomas Jones

DeShauna Jones

Harlan Jones

Yvonne Joosten

A. Kalet

Amrita Kamat

Niranjan Karnik

Elinore Kaufman

Peter Kaufmann

Heidi Keeler

Mary Kate Kelly

Sara Kelly

Denise Kent

Nick Kenyon

David Kern

Mohammad Khalili

Burhan Khan

Lisa Kilpela

Bo Kim

David Kimberlin

Judy A. Kimberly

Robert Kimberly

Jordan King

Elaine Kiriakopoulos

Heather Kitzman

Darya Kizub

Amber Kleckner

Lisa M. Klesges

Kevin Knudtson

Angelica Koch

Natalie Katarzyna Kolba

Ariella R. Korn

Rhonda G. Kost

Melissa Kottke

Jack Kues

Polina Kukhareva

Jonathan Kuo

Matthew Kushner

Sheila Kusnoor

Bethany M. Kwan

Daniel Lackland

Amit Lahoti

Joan Marie Lakoski

Marcus Lambert

Beth LaPensee

Fred Willie Zametkin LaPolla

Bernard LaSalle

Bernie LaSalle

Jeff Laux

Christopher Leckey

MinJae Lee

Rebekka Lee

Suhwon Lee

Colombe Lemire

Chenyu Li

winston Liaw

Nita Limdi

Zhicheng Lin

Michael Linke

Dandan Liu

Lipeng Liu

Mei Liu

Xun Liu

Rebecca Lobb

Tammy L. Loucks

Samuel Louie

Kathryn Macapagal

Jane Mahoney

Sabrina Malone Jenkins

Lucinda Manda-Taylor

Tony Mangino

Mark B. Marchant

Elise Marino

Kris M. Markman

Aditi Martin

Patrick Martin

Karen G. Martinez

Meghan Martz

Hawkins R. Mary

George A. Mashour

Cathy Mathews

Colleen A. Mayowski

Linda McCauley

Joseph McClernon

Russell McCulloh

Melissa McDaniels

Erin McGaffigan

Rachel McGarrigle

Cindy McGeary

Susan McKernan

Christopher McLouth

Emma Anne Meagher

Heta Mehta

Laura Meiners

Jareen Meinzen-Derr

Ryan Melvin

Robin Mermelstein

Will Meurer

Frederick J. Meyers

Tzeyu L. Michaud

Lloyd Michener

Bradley Mills

Nicole Miovsky

Ila Mishra

Dana Mitchell

Edward Siler Monk

Carlton Moore

Justin B. Moore

Travis Moore

Hamidrazi Moradi

Stephanie Morain

Natalia E. Morone

Cynthia Morris

Katie Mosack

Ahmad Mourad

Jessica Mozersky

Lutfiyya Muhammad

Rahma Mungia

Cristina Murray-Krezan

Audrey J. Murrell

Harikrishna Nakshatri

Emmanuel Nartey

Ann Marie Navar

Donald E. Nease

Eric Nehl

Marcel Neunhoeffer

Khoa Nguyen

Mytien Nguyen

Holly L. Nicastro

Jeff Nirscl

Marie K. Norman

Bryant Norton

Perla Nunes

Ucheoma Nwaozuru

Ruth O’Hara

Kinuko Ohneda

Kathleen O’Malley

Minerva Orellana

Robert A. Oster

Debra Oto-Kent

Freida Outlaw

Mustafa Ozkaynak

Marisha E. Palm

Lalitha Parameswaran

Rechelle Paranal

Victoria Parente

Will Parker

Susan R. Passmore

Clara M. Pelfrey

Linda Perez-Laras

Susan M. Perkins

Cynthia Perry

Christine Pfund

Katrina Phelps

Shane Phillips

Patricia Piechowski

Tim Pirolli

Christopher Pittenger

Roy Poblete

Brad H. Pollock

Gina-Maria Pomann

Kathryn Porter

Trish Pruis

Saptarshi Purkayastha

Susan Pusek

Borsika A. Rabin

Thomas Radman

Sivaraman Rajaganapathy

Suhrud Rajguru

Edward Ramos

Jacquelin Rankine

Damayanthi Ranwala

Bryce Reeve

David Resnik

Felice Resnik

Melissa L. Rethlefsen

Alexis Richards

Jennifer Richmond

Jennifer L. Ridgeway

Brad Rindal

Marisa Rinkus

Rocio Rivera-Valentin

Dana Rizk

Betsy Rolland

Lisa G. Rosas

Jason Rosenfeld

Kathryn M. Ross

Stephanie Rowan

Doris Rubio

Caleb J. Ruth

Roy T. Sabo

Elias Samuels

Shannon Sanchez-Youngman

Vidya Sankar

Darlene Santiago

Clarissa Schilstra

Susanne Schmidt

Sarah Schmiege

Margaret Schneider

Robert Schnoll

Joan Antoni Schoenenberger-Arnaiz

Phillip Schulte

Andiara Schwingel

Barbara Segarra

Carolin Sehlbach

Bryce Seifert

Harry Selker

Reimund Serafica

Emily Sheffer Serdoz

Autumn Shafer

Jasmit Shah

Raj C. Shah

Fatemeh Shah-Mohammadi

Pravesh Sharma

Vidya Sharma

Clarissa A. Shaw

Laura Aubree Shay

Jianlin Shi

Cathy Shyr

Mina Silberberg

Tamara Simon

Megan Kasimatis Singleton

Emily Slade

Ben Smarr

Jennifer Smith

Kimberly Smith

Heather Smyth

Rodlescia Sneed

Denise C. Snyder

Olga Solomon

William B. Song

Christine Sorkness

Bailey Speck

Jessica Sperling

Heidi M Spratt

Nicole Stadnick

Justin Starren

Kathleen R. Stevens

Anna Steward

Colleen Stiles-Shields

David M. Stoff

Jan Sullivan

Manu S. Sundaresan

Nicole Sur

Zul Surani

Lynn Sutton

Kera Swanson

Whitney Ann Sweeney

Robert A. Swerlick

Lauren A. Szczygiel

Umberto Tachinardi

Meagan Talbott

Yuan Tang

Marquita Taylor

Sandra Taylor

Dorota Temple

Alexander Testa

Ashley Thomas

Neena Thomas

Beth B. Tigges

Jonathan Tobin

Milan Toma

Sebastian T. Tong

Umit Topaloglu

Sinem Toraman Turk

James Torner

Anna Tosteson

Dustin Tracy

Trevor Tuma

Amalia A. Turner

Elizabeth Turner

Sarah Ullevig

Connie Ulrich

Heatherlun Uphold

Randy Urban

Sangeetha Vadakke-Madathil

Gayle Valensky

Elizabeth Vasile

Francisco Velez

Clare Viglione

Alfred Vitale

Karen Vitale

Adam Vogel

Md Wahid

Anita Walden

Daniel Walker

Nina Wallerstein

Wendy Walsh

Gang Wang

David Warburton

Mary Warner

Judy Washington

Molly Wasko

Jeannette Watterson

Bennett Waxse

Allison Weathers

Kevin Weinfurt

Alexandra J. Weissman

Lisa C. Welch

Leah J. Welty

Jack Westfall

Christine M. Weston

Paris Wheeler

David Whellan

Gretchen E. White

Charles E. Williams, II

Lindia Willies-Jacobo

Jurran Wilson

Karen Wilson

Nicole A. Wilson

Hill Wolfe

Nicole Wolfe

Rebeca Wong

Amanda Woodworth

Theodore Wun

Yaomin Xu

Sharon Yeatts

Jingbo Yi

Alex Yoon

Reza Yousefi Nooraie

Fei Yu

Kexin Yu

Guoyang Zhang

Lixin Zhang

Xuhong Zhang

Yiye Zhang

Yutong Zhang

Shujin Zhong

Emily B. Zimmerman

Kristine Zimmermann

Adam Zolotor

